# The Prevalence of Headache in Crohn's Disease: Single-Center Experience

**DOI:** 10.1155/2016/6474651

**Published:** 2016-01-19

**Authors:** Ulker Anadol Kelleci, Turan Calhan, Abdurrahman Sahin, Resul Kahraman, Kamil Ozdil, Haci Mehmet Sokmen, Destina Yalcin

**Affiliations:** ^1^Umraniye Education and Research Hospital, Department of Neurology, Umraniye, 34764 Istanbul, Turkey; ^2^Mehmet Akif İnan Training and Research Hospital, Department of Gastroenterology, 63300 Şanlıurfa, Turkey; ^3^Umraniye Education and Research Hospital, Department of Gastroenterology, Umraniye, 34764 Istanbul, Turkey

## Abstract

*Objectives*. This study is aimed at studying the prevalence and characteristics of different types of headaches in patients with Crohn's disease.* Materials and Methods*. 51 patients in Crohn's disease group (F/M: 26/25) and 51 patients in control group (F/M: 27/24) were involved. Patients in Crohn's disease group were diagnosed and monitored according to European Crohn's and Colitis Organization diagnostic criteria. The control group composed of healthy subjects with similar age and sex to Crohn's disease group. Headache was classified using the International Headache Society II criteria.* Results*. Headache was reported by 35/51 (68.6%) patients in Crohn's disease group and 21/51 (41.2%) patients in the control group. The prevalence of headache was statistically high in the group with Crohn's disease (OR: 3.125 (95% CI: 1.38–7.04); *p* = 0.01). Comparing two groups with respect to their subtypes of headaches resulted in that the tension-type headache was statistically (*p* = 0.008) higher in Crohn's disease group (26/51) than in the control group (12/51). However, no significant difference was found in the migraine-type headache (*p* = 1).* Conclusions*. This study indicates that the prevalence of headache is high in patients with Crohn's disease and most commonly associated with the tension-type headache.

## 1. Introduction

Crohn's disease (CD) is a lifelong disease caused by interaction between genetic and environmental factors. The exact etiology is currently unknown, so a curative therapy is not available [1]. At present, CD is considered a systemic disease that may be associated with extraintestinal findings as well as intestinal involvement and accompanied by other inflammatory/autoimmune diseases.

The prevalence of extraintestinal manifestations (EIMs) ranges from 6% to 40% in inflammatory bowel disease (IBD) in which many organs including joints, skin, eyes, and hepatobiliary system were involved [2–4]. Some of the EIMs are related to disease activity. EIMs represent the main cause of morbidity in CD [5, 6]. In inflammatory bowel diseases, many neurological complications, including headache, may occur as EIMs, and they are considered to have a prevalence ranging from 0.2% to 47.5% [2, 7–11]. However, those complications may often go clinically undiagnosed and/or the diagnosis may be delayed. The exact etiology of headache and other neurological complications in CD remains unknown, but hypothetically many factors, including immune-mediated phenomena, nutritional deficits, prothrombotic conditions, and use of medications, have been implied [12].

CD is a lifelong disease which usually requires multiple drug use and/or more than one surgery. Additional presence of headache in CD patients significantly impairs the quality of their daily life. The prevalence of headache and its association with the disease still needs to be clarified. Furthermore, early assessment of patients for headache is important in early treatment, compliance, and quality of their life. There is very limited data in the literature for the prevalence of headache in CD patients. The present study aimed to investigate the prevalence, types, and correlation of headache with the disease in Turkish patients with Crohn's disease.

## 2. Materials and Methods

This study was designed as a cross-sectional single-center study. The patient group was selected from patients diagnosed with CD according to European Crohn's and Colitis Organization (ECCO-2010) criteria and followed up at the Gastroenterology Clinic of the Umraniye Education and Research Hospital (Istanbul-Turkey) between January 2012 and December 2012 [1]. The control group included age- and sex-matched volunteers from hospital staff and volunteers from among individuals who admitted to the Gastroenterology Clinic for dyspeptic complaints. Subjects in the control group were individuals not having any inflammatory disease or systemic disorder. Written informed consent was obtained from all subjects. The study protocol was approved by the Ethics Committee of our hospital (number: 14068, 2011).

Physical examinations of CD group were carried out by a gastroenterologist (Turan Calhan) while neurological examinations and headache assessments of all participants in both groups were performed by a neurologist (Ulker Anadol Kelleci). The patients in CD group completed a two-part questionnaire. The first part included questions about demographic characteristics of Crohn's disease, duration of disease, onset age, location of disease, type of disease, and extraintestinal manifestations. The second part was composed of two subsections; the first subsection included all questions required by the International Headache Society (IHS) criteria [13], such as if patients had any headache and, if any, its characteristics (quality, duration, frequency, and location, severity of pain, associated symptoms, aggravating and alleviating factors, and presence of aura). This subsection also evaluated headaches attributed to trauma, substance abuse or withdrawal symptoms, infection, metabolic disease, cervical spondylosis, sinusitis, dental conditions, or other cranial or facial structures. The second subsection focused on whether there was any temporal association between onset of headache and onset of CD symptoms and relationship of headache with extraintestinal manifestations of the disease, Crohn's disease activity index (CDAI), and smoking. In this subsection, the medications used by patients for CD and their effect on the headache were questioned. The subjects in the control group were evaluated for presence of headache, and those who had headache completed the first subsection of the second part of the questionnaire to identify the type of headache. Based on data from patient and control groups, headache was classified according to the IHS criteria. Cranial MRI was performed for all patients with headache.

Based on a preliminary study with 20 subjects each from CD and control groups, the sample size was determined to be at least 48 patients in each group in order to have an 80% power at a level of *α* = 0.05. Statistical analyses were performed by NCSS (Number Cruncher Statistical System), 2007 & PASS (Power Analysis and Sample Size), and 2008 Statistical Software (NCSS LLC, Kaysville, Utah, USA). The data were evaluated using descriptive statistical methods (mean, standard deviation, median, frequency, and ratio) as well as Student's *t*-test for intergroup comparisons of parameters with a normal distribution and Mann Whitney *U* test for intergroup comparisons of parameters without a normal distribution. We used Chi-Square test, Yates Continuity Correction, Fisher's Exact test, and Fisher-Freeman Halton test for comparison of qualitative data. Pearson's and Spearman's correlation analysis was used to evaluate correlations between variables. The results were evaluated in a confidence interval of 95% and at a significance level of *p* < 0.05.

## 3. Results

The study was composed of 51 patients (F/M: 26/25) in Crohn's disease group and 51 individuals (F/M: 27/24) in the control group. The neurological examinations of all subjects were normal. The mean age was 35 ± 12 years in Crohn's disease group versus 35 ± 9 years in the control group. No statistically significant difference was found in gender and age between two groups (*p* = 0.8 and *p* = 0.9, resp.).  Table 1 shows demographic data of the study groups.

According to the evaluation of presence of headache, the prevalence of headache was statistically (OR: 3.125 (95% CI: 1.38–7.04); *p* = 0.01) higher in Crohn's disease group (35/51 (68.6%)) than the control group (21/51 (41.2%)).  Table 2 shows a detailed comparison of both groups with respect to their headache and the subtype of their headache and gender for those with a headache.

Twenty-six patients in Crohn's disease group had a tension-type headache, which was the most common type of headache in this group. 10 patients had infrequent episodic, 9 patients had frequent episodic, and 7 of them had a chronic tension-type headache. When both groups were compared for tension-type headache, the tension-type headache was higher in Crohn's disease group (*p* = 0.008) than the control group. On the other hand, no significant difference was found for migraine-type headache between two groups (*p* = 1). One patient had migraine with aura, and 8 had migraine without aura. Cranial MRI was performed for all patients with headache (*n* = 35); 24 patients had normal cranial MRI. In 11 patients, white matter abnormalities were found in 7 of them who had tension-type headache and 4 of them who had migraine-type headache.

The tension-type headache showed no significant difference between two groups when the disease group and control group were compared for parameters such as quality, location, duration, frequency, and severity of headache, accompanying symptoms, and worsening of headache by physical activity (*p* > 0.05). Evaluation of both groups in tension-type and migraine-type headaches by gender showed that there was no significant difference between two groups (*p* = 0.5 and *p* = 0.5, resp.).

No statistically significant association was found between smoking, presence of extraintestinal involvement, location of disease, type of disease involvement, and presence of headache in Crohn's disease group (*p* = 0.5; *p* = 0.3; *p* = 0.6; and *p* = 0.5, resp.). There was no significant relationship between CDAI and medications used by patients and headache in Crohn's disease group.  Table 3 shows detailed comparison of patients with and without headache in Crohn's disease group by different parameters.

**Figure 1:**
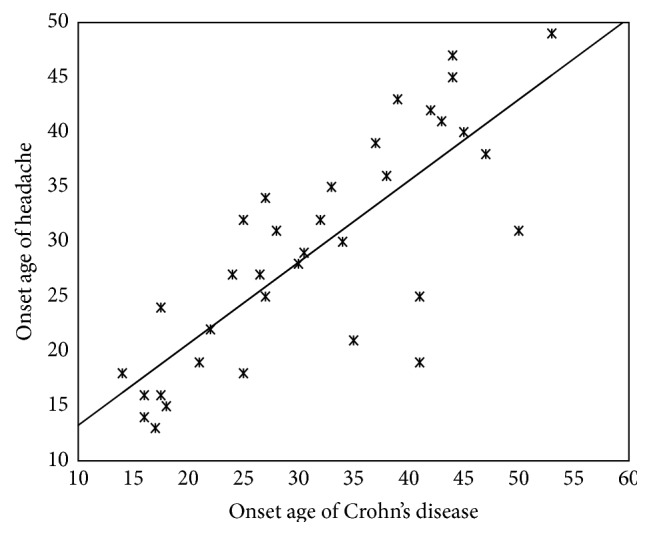
The correlation between the onset age of headache and the onset age of Crohn's disease in Crohn's disease group.

In Crohn's disease group, the onset of headache was after the onset of Crohn's disease in 15 of 26 patients with a tension-type headache and 1 of 9 patients with a migraine-type headache. There was a positive correlation between the onset age of headache and the onset age of Crohn's disease in Crohn's disease group (i.e., the onset age of headache increased as the onset age of Crohn's disease increased) (*r* = 0.7; *p* = 0.001). The mean duration of disease was 29.17 ± 10.24 years in patients in Crohn's disease group.  Figure 1 shows the correlation between the onset age of headache and the onset age of Crohn's disease.

## 4. Discussion

In this study, the prevalence of headache was higher (68.6%) in CD group than the control group. Similarly, a subgroup analysis of headache showed that tension-type headache was also high (51%) in CD group. According to the results of this cross-sectional study, CD was associated with an increased prevalence of headache.

The peripheral and central nervous system complications such as CD and ulcerative colitis (UC) may arise in IBD. However, exact prevalence of these complications is not fully understood [14]. While there are some studies indicating complications are lower with Crohn's disease (2.6%), other studies show high complication rates (33.2%) [7, 8]. Some studies found that CD was associated with cerebrovascular disease, seizures, demyelinating disease, polyneuropathy, mononeuropathy, and myelopathy [7, 8, 10, 11, 14]. Although there are many reports regarding neurological complications in IBD in the literature, the number of reports on headaches associated with IBD is limited. In a study by Elsehety and Bertorini with 253 CD patients, 84 (33.2%) patients had neurological complications and 11 (4.3%) patients had headache, which has been considerably lower than the prevalence in our CD group (68.6%), and, unlike our results, they found that migraine-type headache (7/11) was the most common accompanying headache [8].

Despite limited publications on headache in IBDs, it has been reported that the prevalence of headache was higher in conditions such as systemic lupus erythematosus (SLE), Behçet's disease (BD), and systemic sclerosis (SS), which have been considered to be involved in the etiology of factors such as autoimmune-chronic inflammation. The prevalence of headache was found to be 75.7% by Lessa et al. [15] in patients with SLE, 65% by Haghighi et al. [16] in BD, and 78% by Gökçay et al. [17] in patients with SS. Gökçay et al. discussed the correlation between headache and inflammation, concluding that further studies are needed to clarify this correlation.

Among primary headaches in the population, the most common type is tension-type headache. Although the pathogenesis of tension-type headache remains uncertain, it is most likely to be multifactorial [18]. Environmental factors are considered to have an effect in episodic tension-type headache rather than chronic tension-type headache, while genetic factors play a major role in development of chronic tension-type headache [19–21]. The studies indicate that neurogenic inflammation may be involved in the pathogenesis of primary headaches, although they have a different pathogenesis [22–26]. The onset of headache in one-third of patients with new daily persistent headache during the postinfection period suggests that either headache is triggered by infection or the inflammatory state of the central nervous system is persistent [26, 27]. Koçer et al. [28] found that the serum level of interleukin-6 (IL-6), a proinflammatory cytokine, was higher in patients with tension-type headache compared to the control group, and they discussed the effect of inflammation on the etiology of tension-type headache. Some studies found that levels of cytokines such as IL-1, IL-6, and IL-8 were higher in patients with Crohn's disease [29, 30]. The higher prevalence of headache in CD group in this study may be related to the level of cytokines. However, further studies are needed to explain such a relationship since we did not evaluate the IL levels in our patient group. On the other hand, there was no difference between patients with headache and those without headache according to disease activity. This finding suggests that the chronicity of inflammation is more important than the intensity of inflammation.

Crohn's disease, considered to be involved in the etiology of genetic, environmental, and aberrant immune responses, is a chronic inflammatory disease, and hypothetically headache is most likely to develop as a neurological complication in this immune-mediated setting. Apart from this, at least 6 mechanisms are implied in the etiology of neurological complications in CD: (i) malabsorption and nutritional mechanisms, particularly vitamin deficiencies such as B1, B12, D, E, folic acid, and nicotinamide deficiencies, (ii) metabolic agents, (iii) infections as a complication of immunosuppression, (iv) side effects of medications (metronidazole, sulfasalazine, steroids, and cyclosporine A) or iatrogenic complications of surgery, (v) thromboembolism, and (vi) immunological abnormalities [31]. Although the close temporal relationship between onset of CD symptoms and onset of tension-type headache in some patients in this study may provide support to the claim that inflammation may play a role in the pathogenesis of tension-type headache, the close onset ages of these two conditions may claim the opposite.

This study was carried out in a single center with a limited number of subjects. The focus was mainly on the prevalence rather than pathogenesis of the headache. Although it was a cross-sectional study, the patients completed a questionnaire that aimed to determine onset, prevalence, and type of headache retrospectively, where omission or exaggeration bias should be kept in mind in such studies. Furthermore, while interviewing subjects in both control and disease groups by a single physician contributed to the accuracy for diagnosis of headache, there was no blinding of the interviewer to disease and control groups which was another limitation. Another limitation is about power calculation. Forty-eight patients in each group were found to be sufficient in order to have an 80% power at a level of *α* = 0.05*B* at preliminary study with 20 subjects each from CD and control groups. At the end of the study, post hoc power analysis was carried out with the data obtained from this study by using G^*∗*^Power (v3.1.7) program. Given the distribution of headache in CD group (51 subjects) and in the control group (51 subjects), OR was found to be 3.125 and power was calculated as 82% at a level of *α* = 0.05.

In conclusion, this study indicates that the prevalence of headache is significantly high in patients with Crohn's disease, and it is most commonly associated with a tension-type headache. Similarly, these results show that headache, particularly tension-type headache, should be considered during follow-up of patients with Crohn's disease as a neurological finding, and the treatment should also consider this aspect.

## Figures and Tables

**Table 1:** Comparison of demographic characteristics in both groups.

	CD (*n* = 51)	Controls (*n* = 51)	*p*
	*n* (%)	*n* (%)	
Gender			
Male	25 (49%)	24 (47.1%)	0.8
Female	26 (51%)	27 (52.9%)	
Age (mean ± SD)	35 ± 12	35 ± 9	0.9

CD: Crohn's disease; mean ± SD: mean ± standard deviation.

**Table 2:** Comparison of both groups according to the presence of headache, subtype of headache, and gender for those who had headache.

	CD (*n* = 51)	Control (*n* = 51)	OR	95% CI	*p*
	*n* (%)	*n* (%)			
Headache					
No	16 (31.4%)	30 (58.8%)	3.125	1.38–7.04	**0.01**
Yes	35 (68.6%)	21 (41.2%)			
Subtype of headache					
Migraine-type	9 (17.6%)	9 (17.6%)			1
Tension-type	26 (50.9%)	12 (23.5%)			**0.008**
Subtype of headache by gender					
Migraine					
Male	1 (1.9%)	3 (%5.9)	4.00	0.33–48.65	0.5
Female	8 (15.7%)	6 (%11.8)			
Tension					
Male	13 (25.5%)	4 (7.8%)	2.00	0.48–8.32	0.5
Female	13 (25.5%)	8 (15.7%)			

CD: Crohn's disease; mean ± SD: mean ± standard deviation.

**Table 3:** Detailed comparison of different parameters between patients with and without headache in Crohn's disease group.

	Headache		*p*
	No (*n* = 16)	Yes (*n* = 35)	
	*n* (%)	*n* (%)	
Smoking	3 (18.7%)	11 (31.4%)	0.5
Extraintestinal involvement	3 (18.7%)	13 (37.1%)	0.3
Site of involvement in Crohn's disease			
Ileal	6 (37.5%)	17 (48.6%)	0.6
Ileocolonic	8 (50%)	16 (45.7%)	
Colonic	2 (12.5%)	2 (5.7%)	
Types of Crohn's disease			
Inflammatory	10 (62.5%)	22 (62.9%)	0.5
Fistulating	2 (12.5%)	8 (22.9%)	
Fibrostenotic	4 (25%)	5 (14.3%)	
Medications used			
Mesalazine	16 (100%)	34 (97.1%)	1
Steroid	4 (25%)	5 (14.3%)	0.4
Azathioprine	10 (62.5%)	17 (48.6%)	0.5
Anti-TNF	2 (12.5%)	6 (17.1%)	1
CDAI (Mean ± SD)	73.88 ± 36.99	69.14 ± 33.46	0.6

Mean ± SD: mean ± standard deviation; CDAI: Crohn's disease activity index.
